# The Dilemma of Influenza Vaccine Recommendations when Applied to the Tropics: The Brazilian Case Examined Under Alternative Scenarios

**DOI:** 10.1371/journal.pone.0005095

**Published:** 2009-04-08

**Authors:** Wyller Alencar de Mello, Terezinha Maria de Paiva, Maria Akiko Ishida, Margarete Aparecida Benega, Mirleide Cordeiro dos Santos, Cécile Viboud, Mark A. Miller, Wladimir J. Alonso

**Affiliations:** 1 Evandro Chagas Institute (IEC), WHO Global Influenza Surveillance Network (GISN), Secretary of Surveillance in Health, Brazilian Ministry of Health, Ananindeua, Para, Brazil; 2 Adolfo Lutz Institute (IAL), WHO Global Influenza Surveillance Network (GISN), Secretary of Health of São Paulo State, Brazilian Ministry of Health, São Paulo, São Paulo, Brazil; 3 Fogarty International Center, National Institutes of Health, Bethesda, Maryland, United States of America; University of Liverpool, United Kingdom

## Abstract

Since 1999 the World Health Organization issues annually an additional influenza vaccine composition recommendation. This initiative aimed to extend to the Southern Hemisphere (SH) the benefits—previously enjoyed only by the Northern Hemisphere (NH)—of a vaccine recommendation issued as close as possible to the moment just before the onset of the influenza epidemic season. A short time between the issue of the recommendation and vaccine delivery is needed to maximize the chances of correct matching between putative circulating strains and one of the three strains present in the vaccine composition. Here we compare the effectiveness of the SH influenza vaccination adopted in Brazil with hypothetical alternative scenarios defined by different timings of vaccine delivery and/or composition. Scores were based on the temporal overlap between vaccine-induced protection and circulating strains. Viral data were obtained between 1999 and 2007 from constant surveillance and strain characterization in two Brazilian cities: Belém, located at the Equatorial region, and São Paulo, at the limit between the tropical and subtropical regions. Our results show that, among currently feasible options, the best strategy for Brazil would be to adopt the NH composition and timing, as in such case protection would increase from 30% to 65% (p<.01) if past data can be used as a prediction of the future. The influenza season starts in Brazil (and in the equator virtually ends) well before the SH winter, making the current delivery of the SH vaccination in April too late to be effective. Since Brazil encompasses a large area of the Southern Hemisphere, our results point to the possibility of these conclusions being similarly valid for other tropical regions.

## Introduction

Influenza is one of the most important infectious diseases of humans, and one of the main reasons is that immunity to influenza virus is not life-lasting (as in most of the diseases induced by viruses). This ephemeral period of protection to influenza is due to the fact that the two glycoproteins that protrude from influenza viruses membrane (and therefore the only ones our immune system have access to recognize and “memorize”) have a capacity to mutate frequently, generating varieties different enough to prevent the identification from previous infections, while maintaining their important roles as (among others) binding and releasing sites to the host cells. The natural selection of these viable and rapid antigenic changes in those surface proteins results in the constant necessity of formulating new vaccines for each new influenza season every year. Influenza vaccine effectiveness hence varies each season and depends on how well the vaccine strains match the circulating strains, as well as whether serum antibody response has peaked in the target population before the epidemic arrives [1], [2], [3]. Predicting which strains have the highest probability of circulating in the next season six months ahead (time to produce and distribute the vaccine [2], [4]) based on the study of globally prevailing strains in previous seasons is the challenging task of the World Health Organization (WHO) Global Influenza Program [2], [4], [5].

Since the first annual meeting of the WHO Global Influenza Program in 1973 recommendations have been issued in February, so that vaccines could be made available to the population six months later, before the northern hemisphere winter. Countries in the southern hemisphere only had this recommendation to follow from the WHO, meaning vaccine composition would be determined almost one year in advance of the epidemic season in this region (driving some countries of the Southern Hemisphere -Australia, New Zealand and South Africa- to produce customized vaccines based on their own surveillance [6]).

In Brazil, the inadequacy of such a time lapse became evident already when the first influenza vaccination campaign took place, in the winter of 1998 in the state of Sao Paulo (the campaign was restricted to that state). In that occasion, Brazilian surveillance identified intense epidemic activity of an influenza A/H3N2 strain not included in the northern hemisphere vaccine composition (A/Sydney/5/1997 (H3N2) was circulating whereas the vaccine contained A/Wuhan/359/95 (H3N2)) [6]. In September of the same year WHO issued its first recommendation specifically designed for the southern hemisphere in anticipation of the following winter (June–September 1999), which was then immediately adopted in Brazil in its first nationwide campaign, aimed at providing coverage for the population at highest risk of severe disease outcomes: seniors (>65 years old in the year 1999 and >60 years old since 2000), people suffering from chronic conditions or HIV, as well as health care professionals [6], [7].

In the first three years following its implementation a good match between circulating virus strains and strains included in the recommended composition of the southern hemisphere vaccine was identified (data collected by the Brazilian surveillance project showed 91%,100% and 10% matching between the vaccine composition and the identified strains in 1999,2000, and 2001 respectively [6], although—as we are going to see in this study—timing of circulation relative to vaccine delivery is a critical issue that affects these matching rates). More recently, however, health authorities in Brazil have been discussing the possibility of changing the influenza vaccination calendar for the northern areas of the country due to the perception of the early onset of influenza epidemics in this region [8], [9], [10]—something our results will show to be also relevant to a much wider range of latitudinal zones than has been anticipated until now.

Brazil encompasses a large variety of climates, ranging from equatorial in the North (5.2°N) to subtropical in the South (33.7°S). Previous studies have shown that both the subtropical [6], [11], [12] and equatorial regions of Brazil experience seasonal influenza epidemics, but there is a latitudinal gradient in the timing of the peak of local epidemics, which occurs nearly 3 months earlier (March–April) in the North when compared to southernmost regions [8], [9], [10]. Thus, the viral surveillance data collected regularly over the last decades are of utmost importance to evaluate the effectiveness of vaccination campaigns adopted both in equatorial and tropical regions.

The tropics generally have mild or warm climates and are positioned between the temperate regions where more severe and defined cold seasons alternate each year. Influenza activity in the tropics is more temporally diffuse than the peaks shown at higher latitudes [13], and epidemics display a diversity of seasonal patterns driven by factors not yet clearly understood. For all these reasons, the tropics have less clearly defined seasonality and patterns of trans-hemispheric viral circulation potentially challenging vaccination strategy [7], [13], [14].

**Figure 1:**
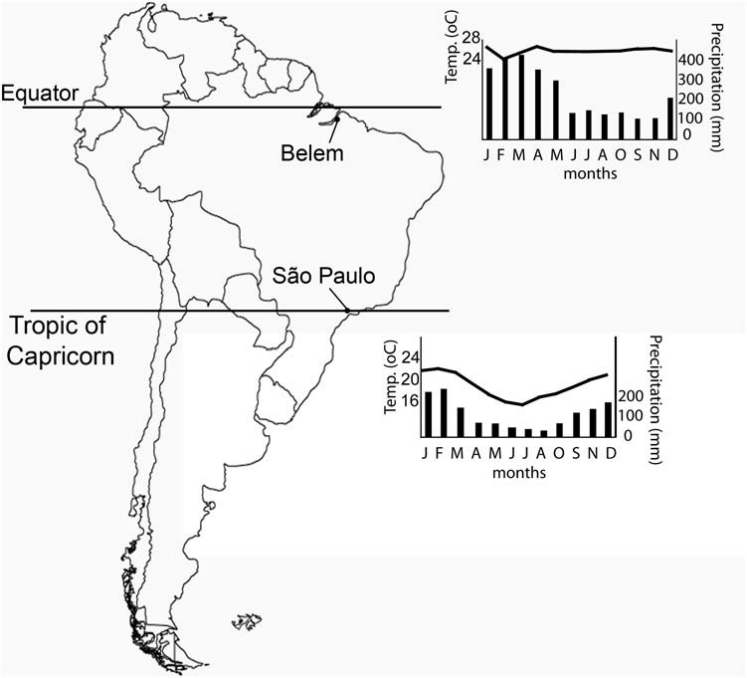
Map of South America, showing the location of Belém and São Paulo, and key monthly climatic indicators in these cities (continuous line represents mean temperatures, and bars represent precipitations). Climatic data are averaged for the period from 1961 to 1990—as made available by the Brazilian Institute of Meteorology [26]

In this study, and based on data from constant surveillance and strain characterization in two Brazilian cities located at the Equator and at the limit between the tropical and subtropical regions (figure 1), we quantify the effectiveness of the influenza vaccination strategy adopted in Brazil and compare it with alternative ones, where vaccination schedule is changed and/or vaccine composition relies on that recommended for the northern hemisphere. “Effectiveness” therefore is operationally defined for the purposes of this study as the proportion of matches between strains present in the vaccine each year and those circulating after the vaccination in the immediately following epidemiological season. By measuring ‘effectiveness’ in this way this study thus accepts the same assumptions about the immunological benefits for the targeted population of a higher match between vaccine composition and circulating strains as those that support the issuing of vaccine recommendations every year for both hemispheres by the WHO.

## Materials and Methods

### Localities studied

The study is based on surveillance of circulating strains identified at two Brazilian cities situated in two places of the tropics of special interest: one is at the core of the tropical region, at a few kilometers from the equatorial line, and the other at its southern boundary – in fact crossed by the tropic of Capricorn line (figure 1).

The city of Belém is located in the Amazonian region at 1.4°S of latitude, and lies in the mouth of the Amazon River. It has an equatorial climate, hot and humid throughout the year, with especially high precipitations during the first half of the year. Belém has approximately 2 million inhabitants and it is the capital of the state of Pará (see climatic profile at figure 1). In its immediate surroundings, in the district of Ananindeua, is located the Evandro Chagas Institute (IEC), a research center for epidemiology and public health from where the Belém data used in this study is originated.

The metropolitan area of São Paulo, crossed by the Tropic of Capricorn, is located at 23.4°S (figure 1) and about 50 kilometers from the coast and 760 meters above sea level. It has a subtropical climate with moderate winters and precipitations concentrated in the warm summer months (see climatic profile at figure 1). With approximately 20 million inhabitants, it is the largest city of the southern hemisphere. Adolfo Lutz Institute (IAL), located in São Paulo, is a National Laboratory on Public Health and a Macroregional Reference Laboratory, and provided us with the material and data from this region.

### Characterization of influenza strains and in Belém and São Paulo

Weekly influenza surveillance is conducted routinely by the two above mentioned Institutes, which also participate at the WHO Global Influenza Surveillance Network (GISN). The sampling effort of this data collection is constant all year round and is part of the influenza national surveillance network of the Brazilian Ministry of Health. It is important to highlight that, although centered in influenza surveillance, this systematic collection of samples also contribute to the investigation of other etiologic agents involved in acute respiratory diseases (which is another reason why the sampling effort is held constant during the entire year). IEC actively sends a technician to collect samples in a local health care center unit near Belém every week. IAL, on the other hand, receives the samples from several sentinel health centers where local physicians have been trained on the detection of relevant cases and on sampling and delivering methods. The samples from São Paulo used in this study are mainly from health centers located in the city of São Paulo, with some samples from localities within a perimeter of 300 kilometers around the city.

Influenza specimens are identified from nasopharyngeal aspirates or swabs taken from outpatients with influenza-like illness. The samples are being tested for Influenza A and B by RT-PCR or Indirect Immunofluorescence, and positive samples are selected for virus isolation and characterized genetically and antigenically following the standard protocol of the Centers for Disease Control and Prevention (CDC, WHO Collaborating Center for Surveillance, Epidemiology and Control of Influenza). Influenza virus isolation (in eggs or MDCK cells) is a laboratory procedure that does not always achieve success (with a positivity rate of approximately 10%). Factors associated with genetic variability of the circulating strains contribute for this occurrence. This explains why so few influenza characterizations are usually available for each country per year (as can be verified at WHO's Flunet website http://gamapserver.who.int/GlobalAtlas/home.asp).

For the two laboratories involved in this study the annual average of virus isolations and characterizations during 1999–2007 (the period encompassed by this study) were, respectively, 2.3 and 2.5 per year at the IEC and IAL Institutes. There was an almost complete matching between the virus strains isolated and those present in the composition of vaccine (produced from extensive surveillance all around the globe) within the same years (Table 1; Figure S1.1), indicating that such sampling effort was indeed representative of circulating virus variants.

**Table 1:** WHO influenza vaccine recommendations for the Southern and Northern Hemisphere, 1999–2007: antigenic characterization of the strains recommended for inclusion in the trivalent vaccine, by hemisphere, influenza season and subtype [16].

**Northern Hemisphere**			
Season	A/H3N2	A/H1N1	B
1998–99	A/Sydney/5/97	A/Beijing/262/95	B/Beijing/184/93
1999–00	A/Sydney/5/97	A/Beijing/262/95	B/Beijing/184/93
2000–01	A/Panama/2007/99	A/New Caledonia/20/99	B/Beijing/184/93
2001–02	A/Panama/2007/99	A/New Caledonia/20/99	B/Sichuan/379/99
2002–03	A/Panama/2007/99	A/New Caledonia/20/99	B/Hong Kong/330/2001 *
2003–04	A/Panama/2007/99	A/New Caledonia/20/99	B/Hong Kong/330/2001
2004–05	A/Fujian/411/2002	A/New Caledonia/20/99	B/Shanghai/361/2002 *
2005–06	A/California/7/2004 *	A/New Caledonia/20/99	B/Shanghai/361/2002
2006–07	A/Wisconsin/67/2005 *	A/New Caledonia/20/99	B/Malaysia/2506/2004
**Southern Hemisphere**			
Season	A/H3N2	A/H1N1	B
1999	A/Sydney/5/97	A/Beijing/262/95	B/Beijing/184/93
2000	A/Panama/2007/99 *	A/New Caledonia/20/99 *	B/Beijing/184/93
2001	A/Panama/2007/99	A/New Caledonia/20/99	B/Sichuan/379/99 *
2002	A/Panama/2007/99	A/New Caledonia/20/99	B/Sichuan/379/99
2003	A/Panama/2007/99	A/New Caledonia/20/99	B/Hong Kong/330/2001
2004	A/Fujian/411/2002 *	A/New Caledonia/20/99	B/Hong Kong/330/2001
2005	A/Wellington/1/2004 *	A/New Caledonia/20/99	B/Shanghai/361/2002
2006	A/California/7/2004	A/New Caledonia/20/99	B/Malaysia/2506/2004 *
2007	A/Wisconsin/67/2005	A/New Caledonia/20/99	B/Malaysia/2506/2004

Asterisks (*) indicate a change in vaccine composition from the previous recommendations.

### Seasonal patterns

In order to contribute to the description of the seasonal patterns of influenza in Belém and São Paulo, we also conducted basic exploratory statistical analyses using the number of influenza isolates aggregated by month and data from 1991 to 2007 (with the exception of 1992 and 1993, when surveillance was interrupted at IEC). We analyzed the frequencies of monthly isolates in both localities using circular (azimuthal) statistics to identify the midpoint of the epidemic season [15]. The concentration of isolates in this time cycle is expressed with the Kappa index, which indicates how the data vectors cluster around the mean (with zero representing evenly dispersed data, hence no seasonal peak) [15].

For comparison purposes, we plotted the average monthly mortality rate from pneumonia and influenza from 1996 to 2005 in each city and surrounding “microregion” as previously described [8], based on data provided by the Brazilian Institute of Geography and Statistics (www.ibge.gov.br/english).

### Analyses of vaccination effectiveness according to different scenarios, 1999–2007

To evaluate and compare the alternative vaccination strategies, we analyzed the time series representing the circulation of different influenza virus strains by subtype and city, relative to the time period when the influenza vaccine would effectively protect the target population. We estimated the proportion of seasons with a correct match of strains circulating and present in the vaccine composition, as defined by the temporal overlap between vaccine-induced protection and circulating strains. Data for the southern and northern hemisphere recommendation can be found on the WHO website [16].

Every year a cohort of people who had never been vaccinated in previous years is vaccinated. Therefore we wanted to determine the effectiveness of the campaign at each single year, minimizing the residual effects of the population already protected for a specific strain at the time of vaccination (i.e. current season) owing to the effect of vaccination in the previous years. Hence, we considered a length of vaccine-induced protection of 9 months following vaccination, to reduce the possibility of considering as a positive match the temporal overlap between (1) a circulating strain in the current epidemiologic season and (2) the immunization to that strain in the immediately previous epidemiologic season. Since 9 months of protection was assumed, the year of 1999 was included because absence of southern hemisphere vaccination in 1998 does not affect the comparison of strategies in 1999 (only if we had detected in January of 1999 a strain already present in the vaccine, but that was not the case). The analyses were conducted independently for each city (so that we could account for the epidemiologic specificities of each locality) followed by an overall analysis considering both cities together.

We first performed our analyses according to an historical vaccine scenario (here referred to as “scenario 1”), i.e. considering those vaccination campaigns that were actually implemented in Brazil, as following to southern hemisphere composition recommendations and schedules. The vaccination campaign is generally conducted during the second half of April and the first week of May and, although the timing of vaccination campaigns varied slightly according to year, we considered that protective vaccine response was achieved by early May.

Secondly, we estimated vaccine match for alternative hypothetical strategies, relying on the Northern Hemisphere vaccine recommendations and/or earlier timings of vaccination (similarly assuming that protective vaccine response was achieved in the month following that of the vaccination campaign). Those hypothetical scenarios are specified below:

#### (scenario 2) Northern hemisphere composition delivered at current southern schedule

This scenario assumes the use of the same composition as that issued for the northern hemisphere and delivery at the same time as the currently adopted schedule for the southern hemisphere (April). Technically, this strategy would be the simplest one, as producers of vaccines for Brazil would be able to know the composition of the vaccine well before (approximately 12 months in advance) the time of delivery.

#### (scenario 3) Southern hemisphere composition delivered three months earlier than the current schedule

This scenario assumes that, from the time the Southern recommendation is issued in September, vaccine manufacturers would have only three months to produce and distribute the vaccine. This is not feasible under current methods of vaccine production, but shortening the time of the production of vaccines has been a priority [17]. We therefore analyze this scenario to evaluate this possibility in case it is made available in a short future.

#### (scenario 4) Northern hemisphere composition as administered in the North hemisphere

Under this scenario vaccines would be delivered using the same composition as that issued for the Northern Hemisphere as well as the same delivery time (November). This scenario therefore checks the matching of circulating strains against vaccine composition as if the Southern Hemisphere recommendation had never existed, with Brazil adopting the same vaccine composition and timing as the countries of the Northern hemisphere (i.e. delivery is assumed thus to occur just before the Southern Hemisphere summer).

The results obtained from these different hypothetical scenarios are summarized in a table. The results of the historical scenario are then compared with those from the hypothetical alternatives with two-tailed chi-square (χ^2^_1_) statistics (contingency tables used historical versus hypothetical scenario; number of matches versus number of non-matches).

### Cross-protection between strains and data aggregation

We considered influenza viral strains based on their hemaglutinin characteristics, as this surface protein is the key component of natural and vaccine-induced immunity. We are aware that cross-protection can make vaccinated individuals immunocompetent to different strains, what would interfere in this quantification of “effectiveness” [3]. Nevertheless we believe this should not affect the validity of our findings because: 1) all scenarios –historic and hypothetical - are equally exposed to this putative effect, hence not representing a bias of the results in any specific direction; 2) here we adopt the same assumptions as those that the WHO adopts to suggest different vaccine compositions each year, therefore considering the complete match a superior strategy than to rely in a putative cross-reaction of the strains considered [3].

Still, and based on the guidelines provided by the WHO [16], we assumed full cross protection for the following strains of influenza B: Hong-Kong/330/2001, Brisbane/32/2002 and Hong-Kong/1434/2002 in the Victoria lineage, and Beijing/184/93 and Yamanashi/166/98 in the Yamagata lineage, and therefore aggregated them accordingly.

Some strains were isolated in more than one month during the same season and in the same city. In such cases, we considered only the month of the first isolation to avoid replication of the same information, and because the effectiveness of the vaccination scenarios is measured according to their capacity to protect the population before the strains begin to circulate in that season.

## Results

### Influenza seasonal patterns in Belém and São Paulo

**Figure 2:**
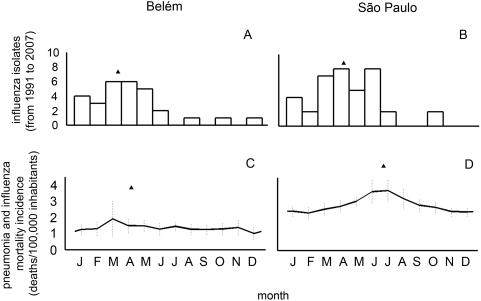
Comparison of influenza seasonal patterns in Belém and São Paulo using laboratory versus vital statistics data. Top panels (A,B) are based on monthly laboratory-confirmed influenza surveillance between 1991 and 2007 Bottom panels (C,D) illustrate monthly mortality rates per 100,000 from pneumonia and influenza (P&I) between 1996 and 2005. Standard deviation are represented as vertical bars. Triangles in each panel represent the mid-point of the influenza season, based on azimuthal (circular) statistics.

During the 1991–2007 period of routine influenza laboratory surveillance, 29 isolates were selected for isolation and characterization in Belém and 38 isolates in São Paulo. Sampling efforts were constant throughout the year, but most influenza viruses were isolated during January and May in Belém, with more activity occurring in March and April (Figure 2A). Similarly, influenza viruses were isolated as early as January in São Paulo, with higher activity occurring in April and June (figure 2b). The average annual timing of influenza isolation, as obtained using azimuthal statistics, was slightly earlier in Belém (end of March, fig 2A) than in São Paulo (end of April, Figure 2B), yet, this difference in timing dispersion was not statistically significant (p = 0.17, i.e. we could not reject the hypothesis that the vector means were the same in São Paulo and Belém [18]). The temporal dispersion around the mean, as measured by the Kappa index, was also similar – in fact slightly lower in São Paulo (1.38) as compared to Belém (1.45).

For comparison with viral activity data, we also explored seasonal patterns in mortality from pneumonia and influenza. Kappa in this case was lower for Belém (0.11) than São Paulo (0.21), suggesting a stronger degree of seasonality in mortality in the latter city , although the dispersion in both cases is quite low, almost uniform, so seasonal effects probably are small in both places. Belém again have shown more intense activity earlier than São Paulo, but this time with a difference of almost three (2.8) months (Figures 2C and 2D), mainly due to the late activity detected in São Paulo in June. Statistical analyses on mortality numbers (without converting them to population ratios) indeed rejected the null hypothesis that the vector mean of mortality were the same in Belém and São Paulo (p<0.001).

Overall, virus surveillance data for this period confirms the seasonal trends evidenced in the analysis of mortality data by suggesting an earlier influenza epidemic activity on average in Belém (although statistical analyses of the viral data could not distinguish the mean annual timing of virus data in Belém and São Paulo with statistical significance).

### Changes in vaccine recommendations between the Northern and Southern Hemisphere

Three antigens are included in the composition of the vaccine every year (A/H3N2, A/H1N1, and B), thus totaling 27 antigens for the 9-year study period from 1999 to 2007. Table 1 lists vaccine recommendations by hemisphere and season during this period. Six changes were first introduced in the Southern Hemisphere recommendations, so that a total of 21 strains (78%) present in the recommended composition for the Southern hemisphere were also present in the recommendations for the Northern hemisphere 6 months earlier. Of the six changes first introduced in the Southern Hemisphere recommendation, 3 were for influenza A/H3N2, 2 for influenza A/H1N1 and 1 for influenza B. During the same study period, 4 changes to vaccine composition were first introduced in the Northern Hemisphere recommendations (2 for influenza A/H3N2, 2 for influenza B).

### Analyses of influenza vaccination effectiveness according to different scenarios, 1999–2007

**Figure 3:**
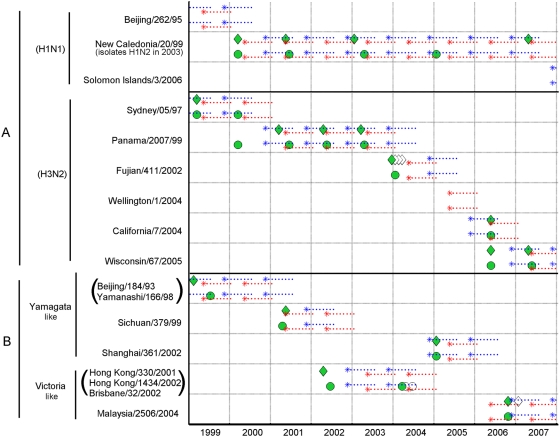
Matching success of vaccine strategies against strains of influenza viruses isolated monthly from 1999 to 2007 in Belém and São Paulo. The different categories of influenza strains considered in the study period are indicated on the vertical axis, sorted by influenza subtype (influenza A) and lineage (influenza B) and identification date. Time is measured on the horizontal axis. Strains isolated each month are represented by green diamonds for Belém, and green circles for São Paulo (blank symbols represent subsequent isolations of the same strain in the same season, and therefore were not considered for the analysis). Stars represent the first month of the period of vaccination-induced protection, while the following dotted lines represent the remaining months of protection. Red lines correspond to historical vaccination strategy adopted by the Brazilian authorities (i.e. relying on the southern hemisphere vaccine recommendations and schedule). Blue lines represent a hypothetical scenario whereby the northern hemisphere vaccination recommendations and schedule are used in both cities. The rate of successful matches between vaccines and circulating strains is quantified by the overlap between vaccine data (blue or red lines, depending on the scenario analyzed) and actual virus isolations (green circles and diamonds) through this period.

The strains of influenza viruses isolated in Belém and São Paulo between 1999 and 2007 are shown in figure 3 by subtype and city, and in Figure S1 (where the process of determining matches between vaccine composition and subsequent circulating strains is explained in detail).

The results showing the effectiveness of vaccination for the different scenarios are shown in Table 2. Cells indicate the number and proportion (in parenthesis) of seasons where strains isolated in São Paulo and Belém were correctly matched (in terms of composition and timing of vaccine delivery) with each vaccination strategy. The total number of strains identified in each location is indicated in the headers (without repetition in the same season, influenza subtype, year and place).

**Table 2:** Success of vaccination campaigns in Brazil for the historical and simulated alternative strategies (1999–2007).

	Vaccination Scenarios	Belém (n = 17)	São Paulo (n = 20)	Overall (n = 37)
Historical	(1) Southern formulation, delivered following southern schedule (April)	5 (29%)	6 (30%)	11 (30%)
Hypothetical	(2) Northern formulation, delivered following southern schedule (April)	3 (18%)	7 (35%)	10 (27%)
	(3) Southern formulation, delivered earlier than southern schedule (January)	12* (71%)	14* (70%)	26** (70%)
	(4) Northern formulation, delivered following northern schedule (October)	11* (65%)	13* (65%)	24** (65%)

* p<0.05 and

** p<0.01: statistically significant difference in the number of positive matches (virus in the vaccine and circulating virus) between the historical and the hypothetical vaccination scenario examined.

Had it been administered in January rather than April (scenario 3 – *Southern hemisphere composition delivered three months earlier than the current schedule* – see “Materials and Methods” and Figure S1.3), the southern hemisphere vaccine would have more than doubled (from 30% to 70%) the proportion of correct vaccine matches with observed influenza epidemics (Table 2; chi-square, p<.01). Surprisingly, the hypothetical scenario relying on the composition and schedule defined by the northern hemisphere recommendations (scenario 4) would have also doubled (from 30% to 65%) the proportion of correct matches (Figure 2; Table 2; chi-square, p<.01).

These results were also statistically significant for each city independently. An unexpected finding is that these results are consistent both for the equatorial city of Belém and for the southern city of São Paulo, which is located on the very transition to the sub-tropical region (figure 1). This finding was consistent for all 3 influenza (sub)types, although weaker for the H1N1 group due to its lower rate of antigenic change during this period.

## Discussion

Under the challenge given by the constant appearance of new influenza strains, delaying for as long as possible the final decision about the composition of the vaccine is without doubt extremely useful, as it increases the chances of successful immunization [2], [3], [4], [5], [6]. Nevertheless, our analysis points to additional complexities that are present in the tropical region, where the concept of ‘hemispheres’ that is so useful for higher latitudes cannot be unequivocally applied.

In this line we have found that, paradoxically, the southern hemisphere vaccination campaign does not stand as the optimal choice for Brazil, the biggest country of the Southern Hemisphere and the second most populated (after Indonesia). In fact, the southern hemisphere recommendations are delivered too late for the whole range of tropical latitudes of Brazil, as the vaccine is offered when the epidemiologic season is already at an advanced stage.

We have described elsewhere [8], [9], [10] that Brazilian regions near the equator had earlier influenza activity than Southern regions. In those lower latitudes precipitation – not low temperatures – has been frequently pointed out as the driving factor [7], [8], [9], [10], [19] facilitating the circulation of influenza viruses - in fact, precipitation is more intense in the first semester of the year in Belém. Accordingly, here we found that the pattern of virus isolation in Belém (Figure 2A) – concentrated in the first half of the year - is consistent with the seasonal patterns of mortality due to pneumonia and influenza in that city (Figure 2C). In São Paulo there was also a major presence of isolates of influenza in the beginning of winter (June), a result expected for a subtropical city with colder winters. But, unexpectedly, we have also shown in Sao Paulo - similarly to the observation for Belém - high levels of influenza virus activity along the first half of the year (Figure 2B). This early activity is not associated with increased pneumonia and influenza mortality (Figure 2A). Indeed, circulation of influenza viruses without significant increases in influenza morbidity or mortality has already been described [20]. It is beyond the scope of this study to seek an understanding of the causes underlying this discrepancy, but we can speculate that intense viral activity in the northern hemisphere winter could drive the early activity in São Paulo (a megalopolis with intense movement of passengers from and to the Northern Hemisphere), while occurrence of severe clinical outcomes following infection may be limited to winter climatic conditions in later months of the year. This could also explain why some studies point to the positive impact of the implementation of influenza vaccination in the South of Brazil [21], [22], while failing to detect such effects in the Northeast [23] – see [7] for a critical review.

In any case, of pivotal importance for the evaluation of the vaccination strategies is the fact that, due to this early viral activity in São Paulo, there was not a significant difference in the overall timing of viral activity (as measured by the vector means of the distribution of the isolates) between both cities. And since both cities essentially shared the same antigenic strains of influenza virus, along with the similar timing of epidemic onset, the effects of the different vaccination strategies were equivalent for both places: one city in the very core of the tropical zone, and another in its Southern geographic limit.

Despite limited resources and a wide range of health related demands, influenza vaccine is delivered free of charge nationwide in Brazil since 1999 to the population at highest risk of severe disease outcomes [6], [7]. Until it is not possible to implement the southern hemisphere recommendation three months earlier than it is currently delivered (the best strategy as pointed by our results), the adoption of the composition and schedule recommended for the northern hemisphere in the northern region of Brazil should be seriously considered. The adoption of such a solution should not present major logistical challenges, as Brazil is attaining self-sufficiency in vaccine production (currently produced by the “Butantan Research Institute”, located in São Paulo).

Tropical regions are increasingly being acknowledged as being of crucial importance for the study of circulation of influenza. In fact, recent studies of the genetic and antigenic characteristics of influenza A virus suggest that the tropics may contain foci for the emergence of new strains [24], [25]. In addition, the tropics also encompass most of the countries with fewer resources for research and health surveillance. Together, these issues hinder the implementation of effective influenza vaccination strategies in the area. The data for this study came from two distant Brazilian cities, representing a large range of climatic, latitudinal, and demographic characteristics. The convergence of results suggests that the relevance of our findings is not restricted to the localities we studied, but could prove pertinent to many other tropical regions of the southern hemisphere. Further studies in tropical regions of Africa, Asia, and Oceania are warranted.

## Supporting Information

Figure S1 This figure illustrates the algorithm used to estimate the proportion of match for different vaccination strategies in Belem and Sao Paulo, Brazil, using data on influenza activity during 1999 to 2007 in these cities and assuming that vaccination protection lasts for 9 months. The different categories of influenza strains considered in the study period are indicated on the vertical axis, sorted by influenza subtype (influenza A) or lineage (influenza B) and identification date. Time is measured on the horizontal axis. Strains isolated each month are represented by green diamonds for Belem, and green circles for Sao Paulo (white symbols mean repeated isolations in that season, therefore not considered). Stars represent the first month of the period of vaccination-induced protection, while the following dotted lines represent the remaining months. Red lines correspond to historical vaccination strategy adopted by the Brazilian authorities (i.e. relying on the southern hemisphere vaccine recommendations and schedule). Blue lines represent a hypothetical scenario whereby the northern hemisphere vaccination recommendations and schedule are used in both cities. Successful matches between vaccine and circulating strains were encircled by hand and counted (partial and total “T = ” counts also shown) originally in these supplementary figures. Data shown in Figures S1.1 and S1.4 are summarized in figure 3 of the main text, and estimates of the proportion of matched seasons for the 4 strategies considered here are given in Table 2.
(0.40 MB DOC)
