# Supplementary material for: The Dilemma of Influenza Vaccine Recommendations when Applied to the Tropics: The Brazilian Case Examined Under Alternative Scenarios
Source: PLoS One. 2009 Apr 8;4(4):e5095. doi: 10.1371/journal.pone.0005095 (PMC2663029; doi:10.1371/journal.pone.0005095)

**These figures illustrate the algorithm used to estimate the proportion of match for different vaccination strategies in Belem and Sao Paulo, Brazil, using data on influenza activity during 1999 to 2007 in these cities and assuming that vaccination protection lasts for 9 months.** The different categories of influenza strains considered in the study period are indicated on the vertical axis, sorted by influenza subtype (influenza A) or lineage (influenza B) and identification date. Time is measured on the horizontal axis. Strains isolated each month are represented by green diamonds for Belém, and green circles for São Paulo (white symbols mean repeated isolations in that season, therefore not considered). Stars represent the first month of the period of vaccination-induced protection, while the following dotted lines represent the remaining months. Red lines correspond to historical vaccination strategy adopted by the Brazilian authorities (i.e. relying on the southern hemisphere vaccine recommendations and schedule). Blue lines represent a hypothetical scenario whereby the northern hemisphere vaccination recommendations and schedule are used in both cities. **Successful matches between vaccine and circulating strains were encircled by hand and counted (partial and total “T=” counts also shown) originally in these supplementary figures.** Data shown in Figures S1.1 and S1.4 are summarized in figure 3 of the main text, and estimates of the proportion of matched seasons for the 4 strategies considered here are given in Table 2

**Figure S1.1)** Southern formulation, delivered in April


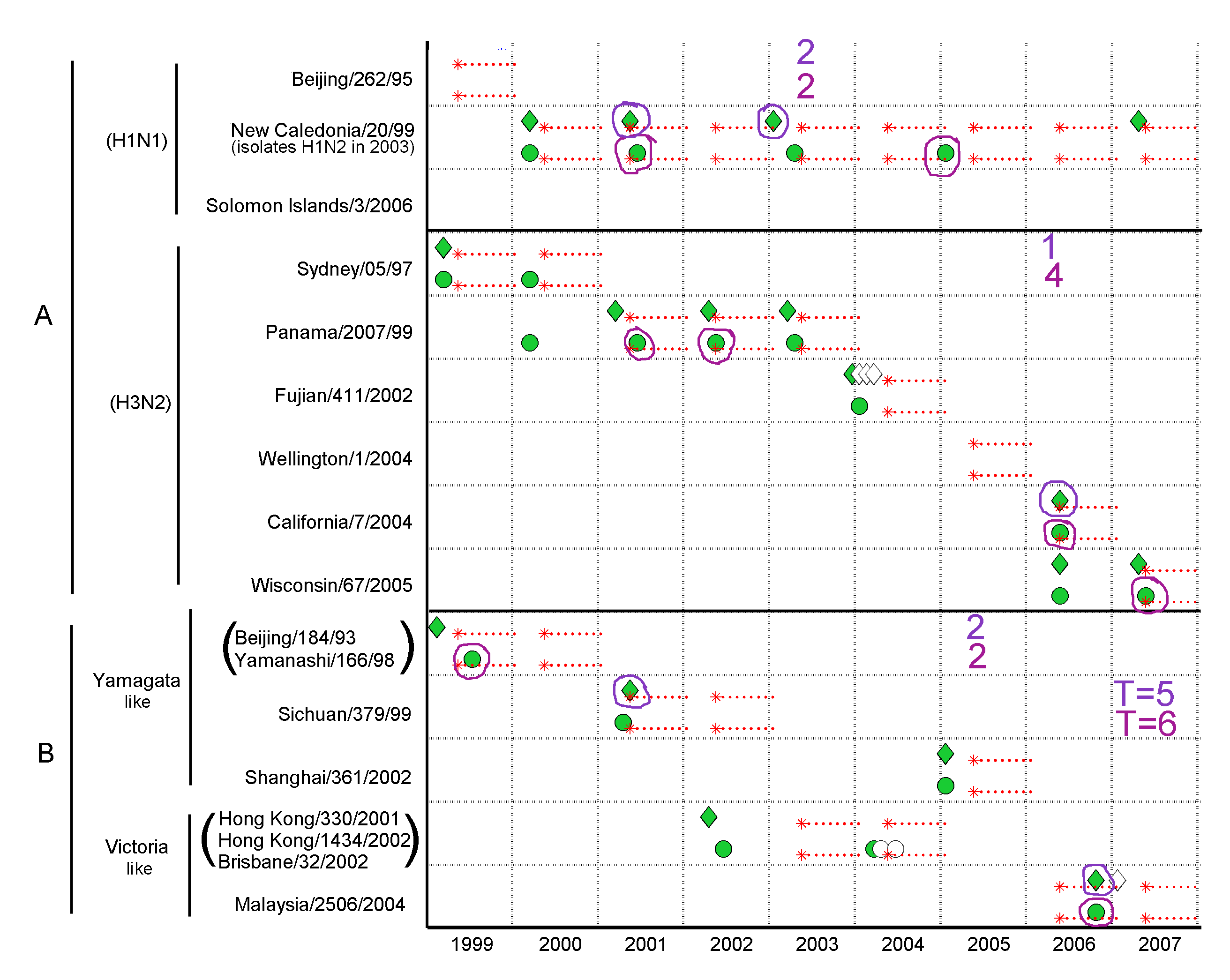


**Figure S1.2)** Northern formulation with Southern timing (April)


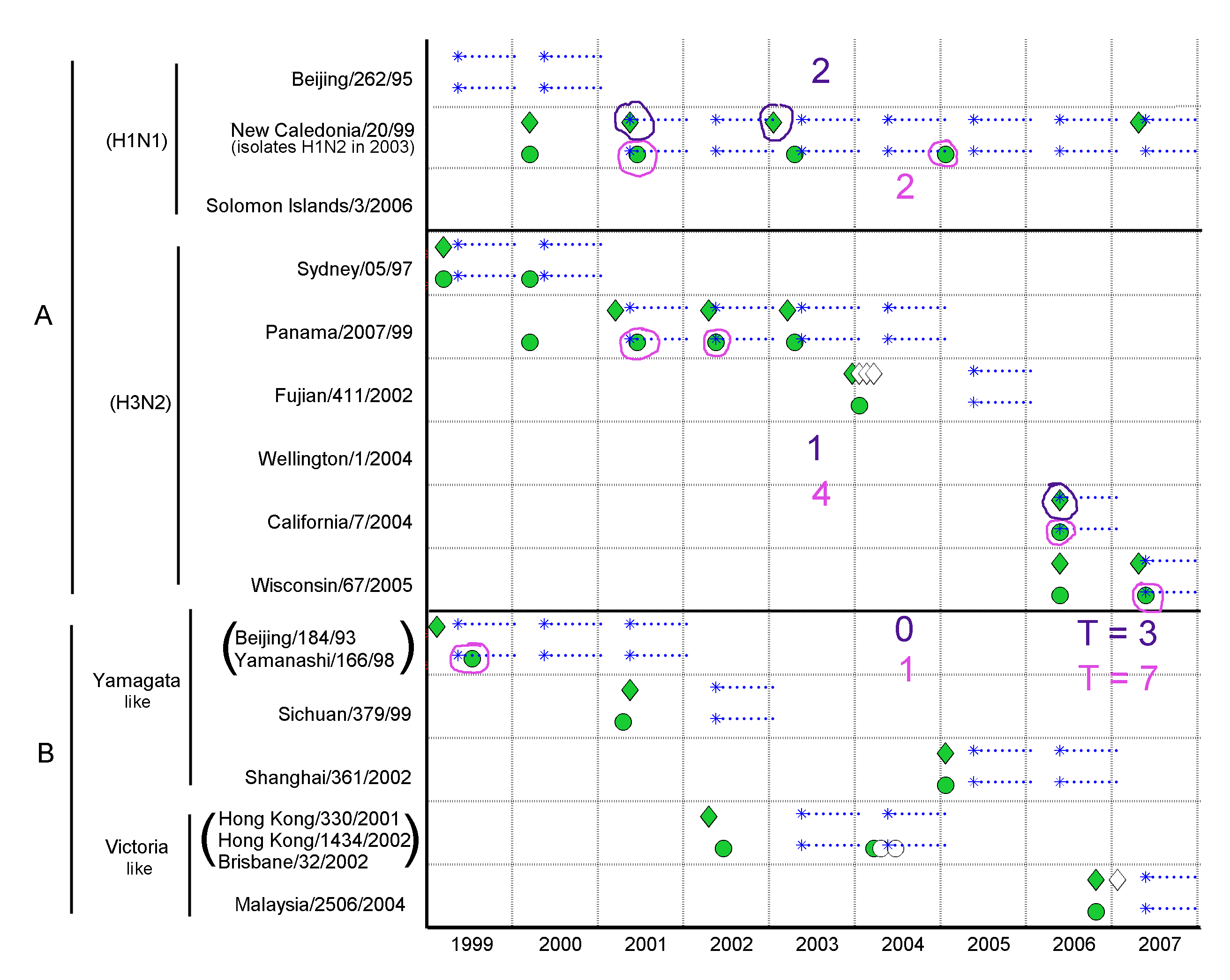


**Figure S1.3)** Southern formulation delivered earlier (January)


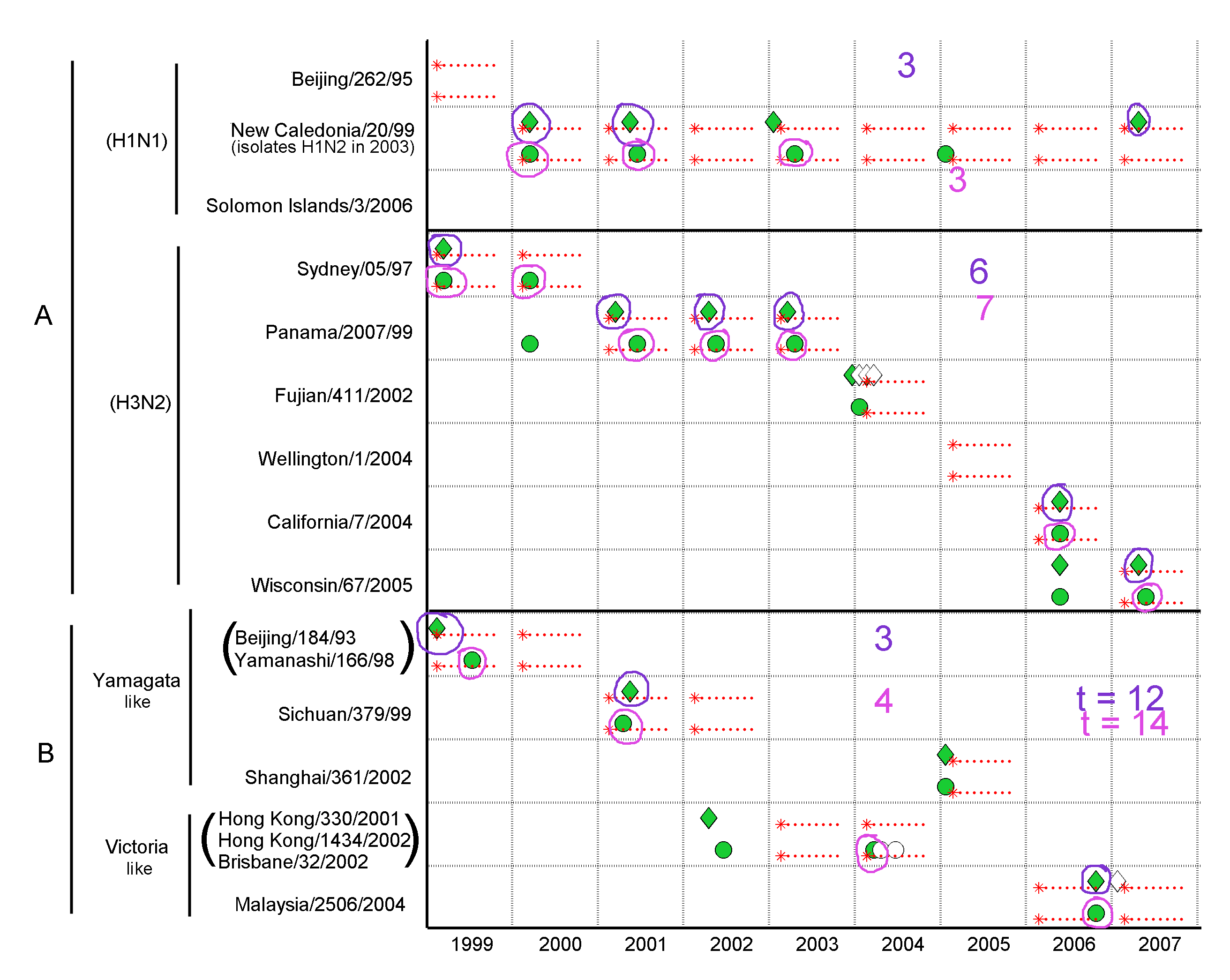


**Figure S1.4)** Identical to Northern hemisphere (Northern formulation delivered in November previous year)


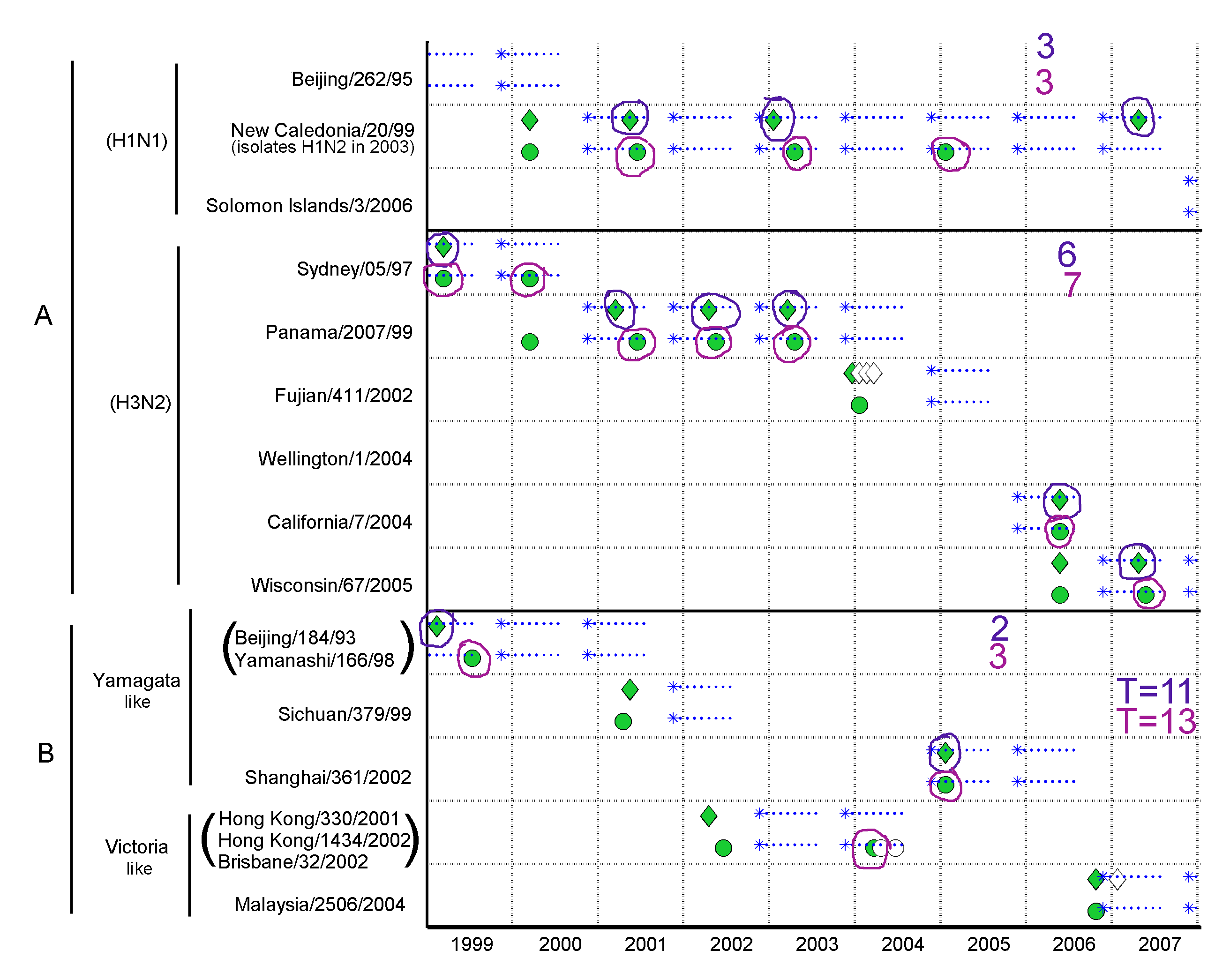

Supplement: Figure S1 — This figure illustrates the algorithm used to estimate the proportion of match for different vaccination strategies in Belem and Sao Paulo, Brazil, using data on influenza activity during 1999 to 2007 in these cities and assuming that vaccination protection lasts for 9 months. The different categories of influenza strains considered in the study period are indicated on the vertical axis, sorted by influenza subtype (influenza A) or lineage (influenza B) and identification date. Time is measured on the horizontal axis. Strains isolated each month are represented by green diamonds for Belem, and green circles for Sao Paulo (white symbols mean repeated isolations in that season, therefore not considered). Stars represent the first month of the period of vaccination-induced protection, while the following dotted lines represent the remaining months. Red lines correspond to historical vaccination strategy adopted by the Brazilian authorities (i.e. relying on the southern hemisphere vaccine recommendations and schedule). Blue lines represent a hypothetical scenario whereby the northern hemisphere vaccination recommendations and schedule are used in both cities. Successful matches between vaccine and circulating strains were encircled by hand and counted (partial and total “T = ” counts also shown) originally in these supplementary figures. Data shown in Figures S1.1 and S1.4 are summarized in figure 3 of the main text, and estimates of the proportion of matched seasons for the 4 strategies considered here are given in Table 2. (0.40 MB DOC) [file pone.0005095.s001.doc]
